# Categorical representation of North American precipitation projections

**DOI:** 10.1038/srep23888

**Published:** 2016-04-04

**Authors:** Arthur M. Greene, Richard Seager

**Affiliations:** 1 Columbia University, International Research Institute for Climate and Society, Lamont Campus, 61 Rte 9W, Palisades, NY 10964, USA; 2 Columbia University, Lamont-Doherty Earth Observatory, Division of Ocean and Climate Physics, 61 Rte 9W, Palisades, NY 10964, USA

## Abstract

We explore use of the familiar tercile framework of seasonal forecasting for the characterization of 21st-century precipitation projections over North America. Consistent with direct analyses of modeled precipitation change, in a superensemble of CMIP5 simulations an unambiguous pattern of shifted tercile population statistics develops as the globe warms. Expressed categorically, frequencies for the low (i.e., dry) tercile increase in the southwestern United States and southward into Mexico and decrease across the northern tier of North America, while counts for the high tercile shift in the opposite sense. We show that as the 21st-century proceeds, changes become statistically significant over wide regions in the pointwise sense, and also when considered as projections on model-specific climate change “fingerprints”. Background noise in the superensemble, against which significance is established, comprises both structural model uncertainty and natural climate variability. The robustness of these findings makes a compelling case for long-range planning for a dryer future in the American Southwest and southward, and wetter one to the north and especially northeast, while communication is facilitated by widespread user familiarity with the tercile format.

Categorical description, in which numerical values are expressed as below, near or above normal, has long been the norm in seasonal forecasting^1^ and is consequently a format familiar to the user community. Terciles, which we utilize here, are simply a special case of description in terms of quantiles, in which a distribution is characterized by the fraction of its probability mass lying below specified thresholds. Thus tercile representation also represents a quantization, and as such, has a smoothing effect, tending to increase signal-to-noise compared with that of the underlying data. For most regions this smoothing increases nominal predictability on the seasonal-to-interannual time scale^2^.

By expressing raw data values in terms of a climate model’s intrinsic distributions, tercile characterization removes biases in both central tendency and spread. However it does not remove biases in regional precipitation sensitivity; the latter are implicitly incorporated into overall uncertainty when ensemble members from different models are pooled, in effect making it more difficult to identify statistically significant changes.

For the 20th century, the climatological period considered here, the probability that rainfall in any year falls in each of the three tercile categories is 1/3, by construction. However as climate warms, rainfall distributions may shift, and with them tercile population statistics (expressed in terms of the climatological category definitions). Given the familiarity and easy interpretability of terciles, casting climate projections in this format should provide the user community with a more readily comprehensible, useful and actionable form of information than presentation in terms of absolute or fractional shifts. Here we present North American precipitation projections in terms of changing tercile probabilities, and show that these probabilities exhibit robust, statistically significant and spatially coherent changes. The changes thus characterized are, as expected, consistent with direct analyses of modeled precipitation changes^3^,^4^,^5^,^6^.

## Results

### Decadal precipitation shifts

#### Terciles vs. anomalies

**Figure 1:**
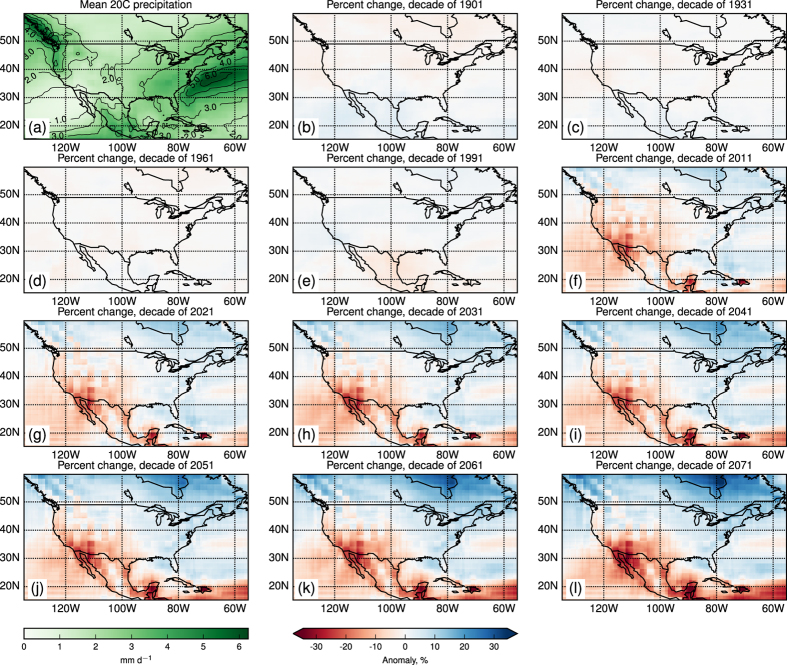
The multimodel mean, annual mean precipitation field (**a**) and anomalies for selected decades spanning 1901–2080 (**b-l**). This figure was generated using Matplotlib version 1.4.3^7^, obtained as part of the Enthought Canopy distribution, see https://enthought.com/products/canopy/.

Panel (a) of Fig. 1 shows the multimodel mean, annual mean 20th-century precipitation field over the study region. (All computations in this report are based on annual mean values.) The remaining panels show decadal-mean anomalies computed with respect to this field, for selected starts spanning the 20th century and for seven decades in the 21st century, the latter beginning with 2011–2020.

Anomalies are relatively weak during most of the 20th century, and of fluctuating sign. However in the decades beginning with 2011 a persistent pattern emerges, consisting of drying in the southwest quadrant and increasing rainfall in the north, east and especially northeast of the map. This pattern, of which a hint may be discerned during 1991–2000, intensifies with time, a response that has been previously identified^8^. The mechanisms responsible for such a shift have also been discussed^9^.

**Figure 2:**
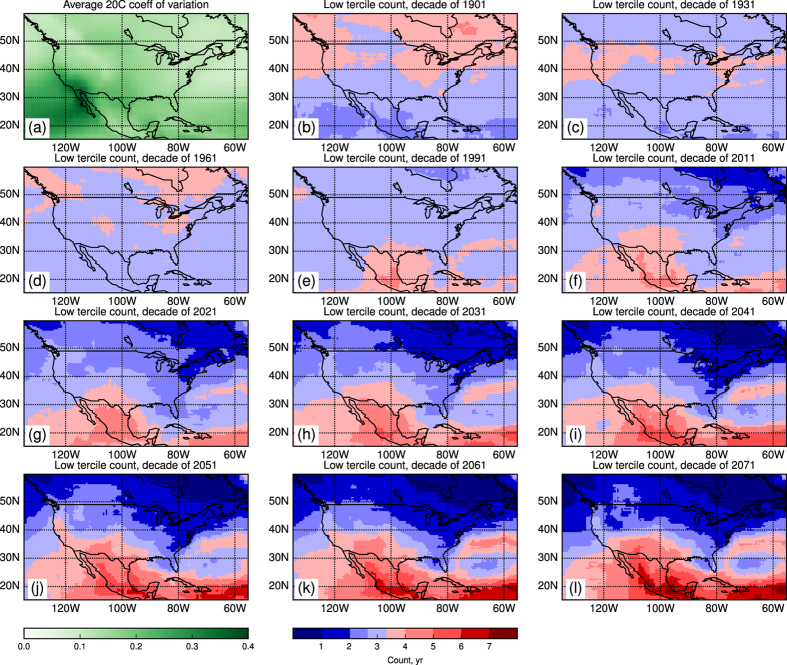
The interannual coefficient of variation, averaged over ensemble members (**a**), and full-decade tercile counts for the same periods as shown in Fig. 1 (**b-l**). The lightest blue and red tones, bracketing the 3.33… climatological value, delimit the approximate 95% confidence interval for no significant change. This figure was generated using Matplotlib version 1.4.3^7^, obtained as part of the Enthought Canopy distribution, see https://enthought.com/products/canopy/.

For comparison, Fig. 2b–k show the same data in terms of decadal counts for the low tercile, i.e., the average across ensemble members of the number of years per decade in which annual mean precipitation falls in the below-normal category. (See Methods re superensemble construction.) The complementary maps, depicting upper-tercile counts, are nearly mirror images, with increased counts where Fig. 2 shows reductions, and vice versa (see Supplementary Fig. S2). Values over the 20th-century maps range mostly from 2.5 to 4 yr decade^−1^, fluctuating around the climatological value of 1/3. However, by mid-21st century, minima less than 1 and maxima between 5 and 6 yr decade^−1^ appear in the northeastern and southern parts of the map, respectively. What was formerly considered a dry year in the latter area is now occurring half of the time or more.

Figure 2a shows the average coefficient of variation, the standard deviation normalized by the mean. The high intensity centered on Baja California and extending into the Pacific is likely a reflection of the low precipitation values characterizing this area (cf. Fig. 1a). The pattern of Fig. 2a does not appear to project strongly onto the emergent pattern of precipitation change.

**Figure 3:**
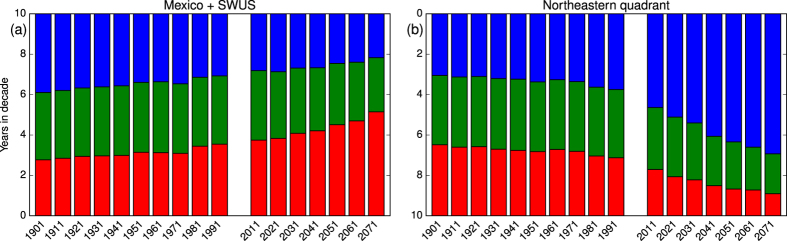
Progression with time of tercile occupation statistics (**a**) for a region that dries (15°–35° N, 90°–120° W) and (**b**) one that moistens (35°–50° N, 65°–80° W). Red, green and blue represent lower, middle and upper terciles, respectively, with years-in-decade represented by bar heights. Y-axis labels are inverted in (**b**) to facilitate assessment of upper-tercile values. Data here are land-only.

Bar plots of area-averaged tercile occupation statistics for regions of relatively intense drying (15°–35° N, 90°–120° W) and moistening (35°–50° N, 65°–80° W) appear in Fig. 3, and show the progression of hydroclimatic change with time. (These data refer to land areas only.) One can see here the approximate symmetry of these two subregional responses, and also that tercile counts gained by the dominating category are lost principally by the opposing tercile, with a contribution also coming from the central category. This is what would be expected from the steady, increasing displacement of a Gaussian distribution across fixed tercile boundaries (see Supplementary Fig. S3 and discussion). These regions are somewhat arbitrary but do represent the two poles of hydroclimatic response.

#### Significant changes

**Figure 4:**
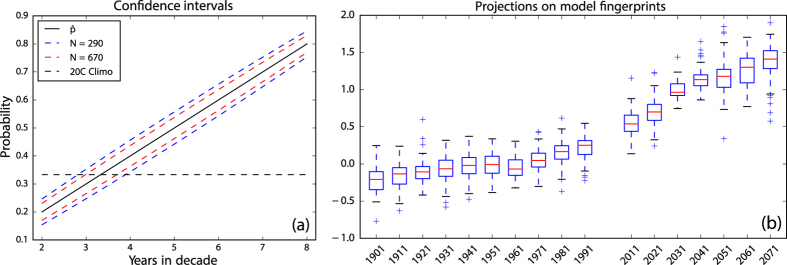
(**a**) Confidence limits (*α* = 0.05) for the number of years in a decade (abscissa) falling in a given tercile, for two values of N. Ordinate shows these values in terms of probabilities. The 20th-century climatological value (1/3) is shown by a horizontal dashed line. (**b**) Distributions of the projection of decadal low-tercile patterns on model-specific fingerprints. Boxes span the interquartile range (IQR), with medians represented by red lines. Whiskers extend 1.5 times the IQR, with any outliers plotted individually.

Figure 4a shows 95% confidence intervals (CI) for the number of years per decade falling in a given tercile, for *N* = 290 and *N* = 670, as derived from the statistics of proportions (see Methods). The higher *N*, representing the number of RCP8.5 ensemble members times the ten years in each decade, would apply if we believed that each ensemble member represented an independent sample of the future climate. The smaller value of *N* (thus wider CI) is based instead on the number of models, and assumes complete dependence among ensemble members from a given model. (There is little evidence of dependence, in the form of year-to-year persistence, within decades.) The climatological probability, 1/3 is shown as a dashed horizontal line; since it has this value by construction it is considered to be perfectly known.

Now consider a shift of the decadal rainfall distributions toward drier conditions, such that precipitation is now falling in the lower tercile five years out of ten, i.e., the probability of a below-normal year has increased to 0.5. Figure 4a shows that even by the “dependent-ensemble-members” measure (smaller *N*), such a shift would be highly statistically significant, with the climatological value (1/3) falling outside the 95% CI for frequencies greater than approximately four years per decade. Shifts larger than this would thus be statistically significant at better than 0.05. Because of the strongly correlated behavior within each of the regions represented in Fig. 3, we may consider them single points. The shifts in lower and upper tercile distributions shown in Fig. 3a,b would then become statistically significant in 2021 and 1991, respectively, using *N* = 670, or 2031 and 2011, using *N* = 290. Note that on the maps of Fig. 2 the approximate 95% CI for no significant change is delineated by the lightest shades of red and blue, bracketing the 3.33… climatological value.

The tercile population shifts depicted in Fig. 2 do not occur randomly over the domain, but exhibit a distinct spatial signature, or “fingerprint”. We define such a climate change fingerprint for each model, as the average of its 2031–2040 and 2041–2050 tercile patterns. Figure 4b shows box-and-whisker plots, by decade, of the distribution of all individual ensemble members’ projections on these fingerprints, expressed as regression coefficients (see Methods).

Consistent with the fingerprint definitions, projections for 2031–2040 and those for 2041–2050 bracket unity. For the 20th century, however, values fluctuate around zero, meaning that decades with inverse fingerprint patterns are about as common as those having positive projections. (Note that values other than for 2031–2040 and 2041–2050 are not constrained by the method of fingerprint construction.) A smooth progression of strengthening projections is evident, with distributions becoming almost completely disjunct from those of the 20th century by the decade of 2031–2040. This represents a statistically significant change in the large-scale spatial pattern of precipitation, expressed in the form of lower-tercile probabilities, and takes into account the possible projection of natural decadal variability onto the climate change fingerprint, within the domains of individual climate models.

Despite a lack of significant shifts during the historical period, projections during this time follow a trajectory suggestive of the global mean temperature record^10^, rising until about 1940, undergoing a gradual decline and then rising again beginning with the 1970s. This “pattern of patterns”, which hints at a dependence of modeled North American hydroclimate on the global mean temperature even during the 20th century, is in fact quite strongly correlated (*r* = 0.99 with N = 10, significant at better than 0.0001, using either the distribution medians or means) with a global mean, decadal multimodel mean temperature record computed from the same ensemble, lending support to this hypothesis.

### Model fidelity

Although the ability of models to realistically simulate the current climate is not a guarantee of future consensus^11^,^12^,^13^, confidence in the results presented may nevertheless be calibrated by a consideration of model fidelity. An extensive analysis would be beyond the scope of the present investigation; rather, we summarize results from several relevant studies.

Assessments have been made of the CMIP5 models’ abilities to simulate both 20th-century climatology^14^ and variability^15^. The models examined in these studies are effectively subsets, utilizing ca. 20 models, of the larger group utilized here. In addition, the period considered for analysis is generally the last 30 yr of the historical model experiments, which extend to 2005. A single ensemble member from each model is considered in most instances. Thus, at a minimum, there are differences with our study across time frame, model population and ensemble structure. Nevertheless there is considerable overlap, in that we depend on the historical simulations to define tercile boundaries and utilize supersets of both models and ensemble members. Thus the results of the cited studies should provide some guidance regarding the degree to which the model simulations on which we rely can be trusted. For purposes of the present study, fidelity in the representation of annual or seasonal mean fields would be most relevant. Pooling or averaging over decades as well as many ensemble members, as we have done, has the effect of attenuating variability, leaving the mean fields. We therefore focus on this aspect of the CMIP5 models.

With regard to large-scale features of the mean precipitation field, and considering both summer (JJA) and winter (DJF) seasons, the multimodel mean was found to represent climatological patterns “reasonably well”, with individual models exhibiting differing degrees of regional bias. Some systematic biases, differing between the two seasons, were also identified. Our expression of climatic shifts in terms of each model’s individual (tercile) climatology would, to first order, remove additive biases at the model level. However to the extent that climatic changes are dependent on the basic state, second-order effects cannot be ruled out. A comparison of 18-model seasonal ensemble means with observations^14^ suggests a reasonable correspondence, particularly when seasonal biases are averaged in the generation of annual means.

With respect to variability, a potentially important finding is that the CMIP5 models tend to do less well at capturing the observed teleconnections. This could present a problem if, say, in the future the El Niño-Southern Oscillation phenomenon (ENSO) shifted toward a preponderance of warm (or cold) events, thereby changing the mean state of the eastern Tropical Pacific. However this was not the case in future ENSO behavior in CMIP5, as reported in a comprehensive analysis of future projections^5^. On the other hand, both the spatial signature and teleconnections of the Pacific Decadal Oscillation (PDO) were found to be reasonably well-reproduced. This was not the case for the Atlantic Multidecadal Oscillation (AMO), however, where teleconnections were generally not well-reproduced. AMO projections have not been well-described, but one study^16^ found that the related Atlantic Meridional Overturning Circulation tended to weaken in the future, without tending toward one phase or the other. This suggests that the lack of fidelity in AMO teleconnections, in the CMIP5 ensemble, does not present a problem as far the present study is concerned.

### Some hydrological qualifications

It is worth keeping in mind that precipitation is only one component in the surface water balance, and it is useful to compare the results presented here with studies that consider other elements as well. These include precipitation minus evapotranspiration, or P-E, a metric comprising both a source and sink^17^, and the Palmer Drought Severity Index (PDSI), closely related to soil moisture availability and considered both in a suite of CMIP3 models^18^ and in the context of paleodroughts^19^. The downscaled hydrology of the western U.S. has also been examined, using the Variable Infiltration Capacity (VIC) water and energy balance model^20^. We focus on precipitation in large part because of its long history as a target variable in seasonal forecasts utilizing the tercile format. However studies such as the above, which provide a higher-dimensional perspective on future water availability, are instructive.

## Summary

We have recast projections of North American hydroclimate, from a large ensemble of climate model simulations, in the tercile format widely used in seasonal forecasting. From the categorical standpoint the projections portray the development of a broadly zonal pattern, with low-tercile counts increasing in the south, including the southwest U.S., and decreasing in the north, particularly in northeastern North America. High-tercile counts exhibit the inverse pattern. Regions where these shifts are statistically significant begin to appear in the final decade of the 20th century and expand with the progression of time. Changes over the domain are highly significant when cast as decadal projections on model-specific climate change fingerprints.

It is hoped that through this transformation the import of projected changes will be better appreciated by decision-makers already accustomed to the interpretation of probabilistic seasonal forecasts. As an example, water managers in the southwest, hoping for a wetter-than-normal year to recharge reservoirs, will comprehend the significance of projections indicating decreasing probabilities for such years, even as the likelihood of dry years increases. Similarly, in the northeast, it is compelling to realize that what was formerly a wet year, occurring one third of the time, by mid-century is projected to occur in some areas an average of six years out of ten. The results presented here demonstrate that the spatial patterns of projected changes are large scale, robust and highly significant. These changes are also socially significant, given the large spatial scale of western water resource systems and agricultural regions.

## Methods

### CMIP5 superensemble construction

We utilize all ensemble members from 29 global climate models participating in the Coupled Model Intercomparison Project, Phase 5 (CMIP5) having both 20th-century (“historical”) and Representative Concentration Pathway 8.5 (“RCP8.5”) simulations. (A complete listing is provided as Supplementary Table S1.) These span the historical (here 1901–2005) and future (2011–2080) periods, respectively. There are a total of 103 historical and 67 RCP8.5 simulations. We show results based on equally weighting all ensemble members, which is equivalent to weighting models differentially depending on ensemble size. Computations based on equally weighting the models, irrespective of the number of ensemble members, result in somewhat greater uncertainty bounds for establishing pointwise significance, but differences are small and do not affect the conclusions presented. (See Supplementary Fig. S1 and associated discussion.)

Large ensemble sizes reduce sampling uncertainty. For example, the occurrence of a single decade in which precipitation falls in the lowest tercile in four years would not be considered evidence of a climatic change; such fluctuations are not uncommon in the historical record. A sample of 67 decades having a low-tercile hit rate of 0.4, however, would provide much stronger evidence that the rainfall distribution had indeed changed. This is effectively the case with the many ensemble members utilized here, but with the sampled decades running concurrently rather than consecutively.

Tercile statistics for an individual model can be computed by pooling ensemble members, since changes are all referred to a common basic state. However combining results across models involves averaging, a procedure that presents complex issues of model inheritance^21^ and interdependence^12^,^22^. We do not explore these matters in detail here, but note that for the decadal time horizons considered, natural internal variability within even a single model may produce a very wide range of responses^23^. Such variability would tend to reduce dependence within the superensemble.

### Computation

From each model we obtained variable “pr” from all available ensemble members, for both the historical and RCP8.5 experiments^24^. Historical runs for 1901–2005 for each model were pooled in order to compute climatological tercile boundaries; RCP8.5 runs for each model were similarly pooled to compute future tercile occupation statistics, based on those climatological boundaries. Probabilities were subsequently averaged over models in order to generate the maps of Fig. 2.

Tercile boundaries at each gridbox were computed as scores at the 33.3… and 66.6… percentiles of each model’s historical ensemble. We show maps and analysis for the lower tercile, in part because an underlying focus is the potential drying, with global warming, of the American southwest. Plots for the upper tercile are almost mirror images, as suggested by Fig. 3, while those for the central tercile for the most part show weaker, quasi-random patterns. As suggested by Fig. 3, increases in the low tercile count are compensated by reductions in both the central and upper terciles, and vice versa (see Supplementary Figs 2 and 3 and associated discussion.) Because of this mirroring of the low by the high tercile we may consider the former alone without loss of generality.

Decadal statistics are computed in each model’s domain by comparison with the climatological boundaries. Since the sample size for an individual model is relatively small, population statistics are computed by first fitting a Gaussian to the pooled ensemble values^25^. Tercile statistics are then averaged over models, weighting each model by the number of its ensemble members (or, for the comparison discussed in the text, equally). It is these multimodel shifts that are shown in Fig. 2. The mean precipitation field and anomaly plots of Fig. 1 are computed on the “raw” model data, giving each ensemble member equal weight; Fig. 2a is also an average over ensemble members.

The confidence intervals of Fig. 4a are computed using the standard statistical measure of uncertainty for proportions^26^:


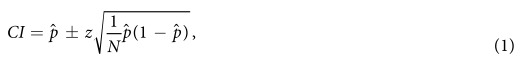


where *z* is the half-width of the desired CI, expressed in standard deviations, 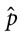 is the estimated probability of precipitation falling in the given tercile and N represents degrees of freedom, or number of independent pieces of information contributing to the estimate. Here *z* is taken as 1.96, corresponding to the 95% CI.

### Climate change fingerprints

For the box-and-whisker plots of Fig. 4b a fingerprint was computed for each model and the model’s individual ensemble members projected on it, decade by decade, yielding the coefficient values summarized in the plot. The projections were computed by reshaping both the fingerprints and decadal maps as *M* × 1 vectors, then regressing each map in turn on the fingerprint. Identical patterns would yield a regression coefficient of unity, while a pattern identical in form but having only one tenth the amplitude would have a coefficient of 0.1. The regression framework thus combines pattern resemblance and intensity, and for this reason was deemed more useful than the use of correlation. Figure 4b was computed in the tercile domain.

## Additional Information

**How to cite this article**: Greene, A. M. and Seager, R. Categorical representation of North American precipitation projections. *Sci. Rep*. **6**, 23888; doi: 10.1038/srep23888 (2016).

## Supplementary Material

Supplementary Information
