# Supplementary material for: Categorical representation of North American precipitation projections
Source: Sci Rep. 2016 Apr 4;6:23888. doi: 10.1038/srep23888 (PMC4819218; doi:10.1038/srep23888)
Supplement: Supplementary Information [file srep23888-s1.pdf]

Supplementary Information

Categorical representation of North American precipitation projections

Arthur M. Greene[1,*] **and Richard Seager**[2]

[1]Columbia University, International Research Institute for Climate and Society, Lamont Campus, 61 Rte 9W, Palisades, NY 10964
[2]Columbia University, Lamont-Doherty Earth Observatory, Department of Ocean and Climate Physics, 61 Rte 9W, Palisades, NY 10964
[*]amg@iri.columbia.edu

ABSTRACT

We present here ancillary information for the above-referenced article, including a listing of the climate models utilized in the analysis. This material is appropriately referenced in the main document text.

Multimodel ensemble

A list of the climate models utilized, along with the number of ensemble members available for each of the historical and RCP8.5 experiments, is provided in Table S1.

Model averaging

We compute a multimodel mean response in two ways, weighting equally either ensemble members or models (alternatively, weighting models according to the number of ensemble members or equally). Figures S1a and S1b show the results of the two procedures for the 2031-2040 decade, while Figure S1c shows the difference field. Differences are not entirely random, showing weak negative values in a band across the central and southern areas of the domain, with positive values at higher and at some lower latitudes. Evidently, models having more ensemble members have a small dry bias as compared with the others: They tend to make the drying areas, coarsely defined, a bit drier and moisten the moistening areas a bit less. In any case the maps of Figures S1a and S1b are quite similar; we judge the difference as being too small to weigh on the conclusions presented.

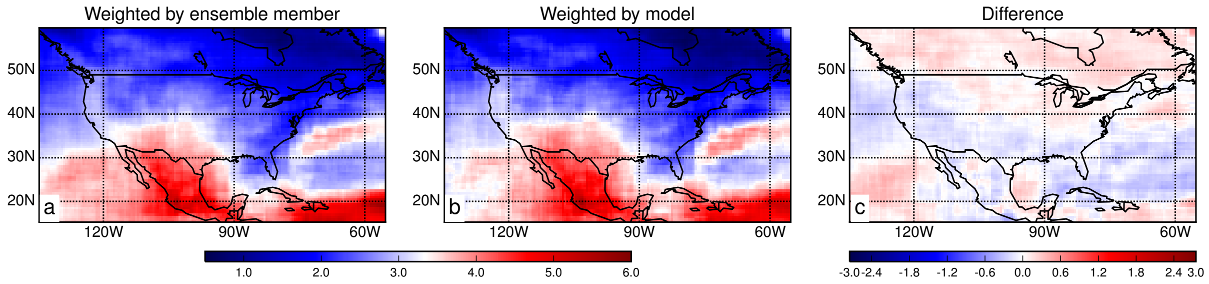

Figure S1. Lower-tercile population statistics for 2031-2040. (a) Values from equally weighting ensemble members. (b) Values from equally weighting the models. (c) Difference field (a minus b). The range in (c) has been set equal to the ranges in (a) and (b) but is centered on zero, rather than the climatological value of 1/3. This figure was generated using Matplotlib version 1.4.3,[1] obtained as part of the Enthought Canopy distribution, see `https://enthought.com/products/canopy/`.

Symmetry of low- and high-tercile counts

Figure S2 shows tercile counts for all three categories for the 2031-2040 decade, and illustrates the approximate symmetry between low and high terciles: Regions where the dry category becomes more frequent correspond closely to those where the wet category becomes less so, and vice versa.

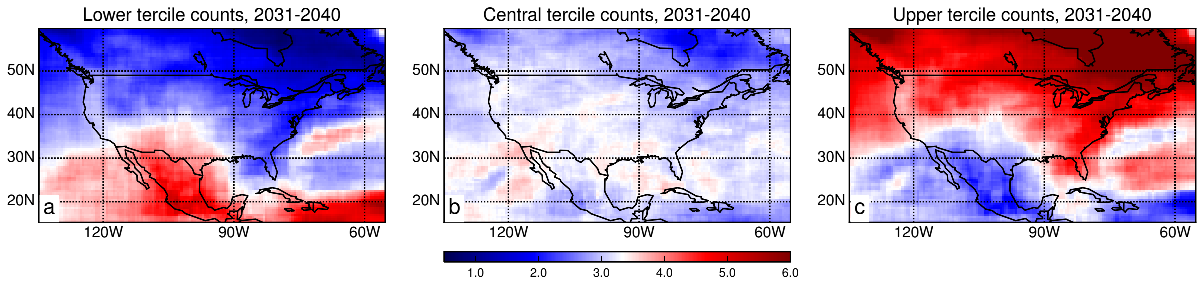

Figure S2. Tercile counts for the 2031-2040 decade. Low, middle and upper tercile categories are shown in panels (a), (b) and (c), respectively. This figure was generated using Matplotlib version 1.4.3,[1] obtained as part of the Enthought Canopy distribution, see `https://enthought.com/products/canopy/`.

Although this relationship may seem like a logical necessity, it does not take into account the behavior of central-tercile probabilities, which in principle could absorb a greater or lesser proportion of the counts lost by the category being depleted. But in fact this migration of counts is approximately constrained: Since the distributions of precipitation anomalies that ultimately generate the counts are approximately Gaussian (these are distribution over both years and ensemble members), the pattern by which counts shift tends to behave as if governed by an underlying, shifting normal distribution.

Figure S3 shows in schematic form how these shifts would play out, using a Gaussian climatological distribution with zero mean and unit variance. Before any climatic change has occurred, all three terciles have equal probability ($1/3$), represented on the abscissa at value zero. As the future distribution shifts leftward, corresponding to drier and drier conditions, the low-tercile probability increases, while probabilities for the others both decrease, the wet-tercile probability falling more than that of the central tercile. This is essentially the situation we see in Fig. 3 (main manuscript), particularly for the northeastern quadrant.

One might argue that since the computation of tercile probabilities is here performed by first fitting Gaussians to the raw model scores, such a result is foreordained. However the fitting procedure serves only to smooth what are already quasi-normal distributions, the Gaussian character existing a priori.

References

1. Hunter, J. D. Matplotlib: A 2D graphics environment. *Computing In Sci. & Eng.* **9**, 90–95 (2007).

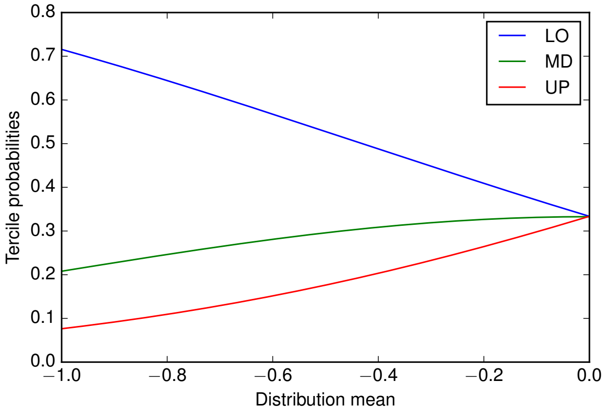

Figure S3. Shifts of tercile probabilities with drying. “LO”, “MD”, and “UP” refer to lower, middle and upper terciles, respectively.

Table S1. Climate modeling centers and models, listed alphabetically by the latter. “hist” and “RCP8.5 columns show number of ensemble members for each model, for the two experiments.

Center	Model	hist	RCP8.5
Commonwealth Scientific and Industrial Research Organization (CSIRO) and Bureau of Meteorology (BOM), Australia	ACCESS1-0	1	1
—	ACCESS1-3	1	1
Beijing Climate Center, China Meteorological Administration	BCC-CMS1.1	3	1
—	BCC-CMS1.1(m)	3	1
Canadian Centre for Climate Modelling and Analysis	CanESM2	5	5
National Center for Atmospheric Research, USA	CCSM4	6	6
Centre National de Recherches Météorologiques / Centre Européen de Recherche et Formation Avancée en Calcul Scientifique	CNRM-CM5	10	5
Commonwealth Scientific and Industrial Research Organization in collaboration with Queensland Climate Change Centre of Excellence	CSIRO-Mk3.6.0	10	10
LASG, Institute of Atmospheric Physics, Chinese Academy of Sciences and CESS,Tsinghua University	FGOALS-g2	5	1
—	FGOALS-s2	3	3
The First Institute of Oceanography, SOA, China	FIO-ESM	3	3
NOAA Geophysical Fluid Dynamics Laboratory, USA	GFDL-CM3	5	1
—	GFDL-ESM2G	3	1
—	GFDL-ESM2M	1	1
NASA Goddard Institute for Space Studies, USA	GISS-E2-H	5	1
—	GISS-E2-R	6	1
Met Office Hadley Centre (additional HadGEM2-ES realizations contributed by Instituto Nacional de Pesquisas Espaciais)	HadGEM2-CC	1	3
—	HadGEM2-ES	4	4
Institute for Numerical Mathematics, Russian Academy of Sciences	INM-CM4	1	1
Institut Pierre-Simon Laplace, France	IPSL-CM5A-LR	5	4
—	IPSL-CM5A-MR	1	1
—	IPSL-CM5B-LR	1	1
Atmosphere and Ocean Research Institute (The University of Tokyo), National Institute for Environmental Studies, and Japan Agency for Marine-Earth Science and Technology	MIROC5	4	3
Japan Agency for Marine-Earth Science and Technology, Atmosphere and Ocean Research Institute (The University of Tokyo), and National Institute for Environmental Studies	MIROC-ESM	3	1
—	MIROC-ESM-CHEM	1	1
Max-Planck-Institut für Meteorologie	MPI-ESM-LR	3	3
—	MPI-ESM-MR	3	1
Meteorological Research Institute, Japan	MRI-CGCM3	3	1
Norwegian Climate Centre	NorESM1-M	3	1
